# Excessive consumption of mucin by over-colonized *Akkermansia muciniphila* promotes intestinal barrier damage during malignant intestinal environment

**DOI:** 10.3389/fmicb.2023.1111911

**Published:** 2023-03-02

**Authors:** Shuang Qu, Yinghui Zheng, Yichun Huang, Yicheng Feng, Kunyao Xu, Wei Zhang, Yawen Wang, Kaili Nie, Meng Qin

**Affiliations:** College of Life Science and Technology, Beijing Advanced Innovation Center for Soft Matter Science and Engineering, Beijing University of Chemical Technology, Beijing, China

**Keywords:** intestinal diseases, *Akkermansia muciniphila*, intestinal barrier, gut microbiota, mucin

## Abstract

Gut microbiota disorders damage the intestinal barrier, which causes intestinal disease. Thus, we screened the microbiota with significant changes using an *in situ* malignant colorectal cancer (CRC) model. Among the colonies with increased abundance, *Akkermansia muciniphila* (*A. muciniphila*) is known for its characteristic of breaking down mucin, which is an essential component of the intestinal barrier. The role of *A. muciniphila* remains controversial. To investigate the effect of excess *A. muciniphila* on the intestinal barrier, we established an over-colonized *A. muciniphila* mouse model by administering a live bacterial suspension after disrupting the original gut microbiome with antibiotics. The results showed that over-colonization of *A. muciniphila* decreased intestinal mucin content. The mRNA and protein expression levels of tight junction proteins also decreased significantly in the over-colonized *A. muciniphila* mouse model. Our findings reveal that excess colonization by *A. muciniphila* breaks the dynamic balance between mucin secretion and degradation, reduces the thickness of the intestinal mucus layer, and damages the intestinal barrier, which would eventually aggravate the development of colitis and CRC. These results will raise awareness about the safety of *A. muciniphila* serving as a probiotic.

## 1. Introduction

A strong association has been reported between the gut microbiota and various diseases, particularly intestinal diseases, including inflammatory bowel diseases (IBD), such as Crohn’s disease; (Palm et al., 2014), ulcerative enteritis (Kummen et al., 2017), and irritable bowel syndrome (Liu et al., 2017), as well as colorectal cancer (CRC; Coker et al., 2022). Among them, CRC has high incidence and mortality rates worldwide (Sung et al., 2021). Since metagenomics next-generation sequencing technology has been developed, researchers are discovering the relationships between the gut microbiota and intestinal diseases (Rajagopala et al., 2017). The complex and extensive microbiota that colonizes the gut is an essential part of the intestinal contents and plays a crucial role in many physiological processes, including immunological control and the regulation of digestion and metabolism (Schultz et al., 2017; Zheng et al., 2020). Accordingly, dysbiosis of the gut microbiota is a major contributor to intestinal diseases (Mahalhal et al., 2018; Wong and Yu, 2019; Lee, 2021). The gut microbiota change in quantity and composition with changes in food intake, lifestyle, use of medications, and other factors, which raises the risk of intestinal disease. The intestinal barrier, which stops invasion by pathogenic microorganisms, includes the gut microbiota as the key component. The intestinal barrier system is damaged by dysbiosis of the gut microbiota, excess colonization of pathogenic microbiota, excess consumption of mucus, and thinning of the intestinal mucus layer, which expose the intestinal epithelial cells to the microbial environment (Van der Sluis et al., 2006; Johansson et al., 2008; Kim and Ho, 2010). Inflammation develops from bacteria directly contacting epithelial cells and is exacerbated by the toxic byproducts produced by pathogenic bacteria. Inflammation is a major contributor to intestinal disease and CRC.

The intestinal mucus layer is mainly composed of mucins. The degradation of mucins is an adverse factor that may lead to disturbances in the host intestinal environment (Paone and Cani, 2020). The intestinal goblet cells continuously secrete mucin, which provides colonic bacteria with a plentiful energy supply to survive (Ouwehand et al., 2005). Some intestinal microbiota have a slow degradation effect on mucin, such as *Akkermansia muciniphila* (*A. muciniphila*; Sicard et al., 2017). *Akkermansia muciniphila* is a Gram-negative, strictly anaerobic bacterium that inhabits the human intestine, and is a representative genus in *Verrucomicrobia* (Derrien et al., 2004). *Akkermansia muciniphila* uses mucin as its sole source of carbon and nitrogen, and produces 61 enzymes that destroy mucin (Derrien et al., 2004). A decrease in the abundance of *A. muciniphila* was thought to be associated with obesity, diabetes mellitus type 2, nonalcoholic fatty liver disease, and cardiovascular diseases (Everard et al., 2013; Zhang et al., 2013; Everard et al., 2014; Li et al., 2017). However, studies on *A. muciniphila* in colitis and CRC are controversial, as *A. muciniphila* promotes mucin secretion and reduces intestinal permeability through extracellular vesicles (Chelakkot et al., 2018). It has also been shown that *A. muciniphila* has beneficial effects on intestinal barrier function during intestinal inflammation. For example, *A. muciniphila* helps to restore intestinal barrier function in DSS-induced colitis, improve the symptoms of colitis, and repair intestinal barrier damage (Kang et al., 2013; Zhai et al., 2019; Wang et al., 2020). However, depleting the mucus layer may be exacerbated by the consumption of mucin by *A. muciniphila*, leading to thinning of the mucus layer and increased inflammation. *Akkermansia muciniphila* interferes with reconstruction of the intestinal mucosa and aggravates the symptoms of colitis caused by *Salmonella typhimurium* (Ganesh et al., 2013). Other studies have demonstrated that *A. muciniphila* acts as a pathobiont to promote colitis in a genetically susceptible host. Our previous study reported an overgrowth of *A. muciniphila* in DSS-induced acute ulcerative colitis (Huang et al., 2022). However, it remains unclear whether this is compensatory regulation of the intestinal microbiota or a pathogenic effect of *A. muciniphila*. In addition, when intestinal inflammation progresses to CRC, the intestinal barrier is completely disrupted, and colonization of *A. muciniphila* is rarely reported at that time.

In this study, we explored changes in the intestinal microbiota of an *in situ* malignant CRC model and investigated the effect of over-colonized *A. muciniphila* on the intestinal barrier.

## 2. Materials and methods

### 2.1. Reagents

CT26 cells were purchased from Procell Life Science & Technology Co., Ltd. (Wuhan, China). Roswell Park Memorial Institute (RPMI) 1640 medium and fetal bovine serum (FBS) were purchased from Gibco (Shanghai, China). Ampicillin sodium salt, neomycin sulfate, metronidazole, and vancomycin HCl were purchased from Macklin Biochemical Co., Ltd. (Shanghai, China). *Akkermansia muciniphila* was obtained from the BeNa Culture Collection (Henan Province, China). The thioglycollate medium was purchased from Qingdao Hope Bio-Technology Co., Ltd. (Qingdao, China).

### 2.2. Animals

The animal experiments used 20–22 g male BALB/C mice provided by the Beijing HFK Bioscience Co., Ltd. (Beijing, China). All mice were raised under standard laboratory conditions with a 12/12 h dark/light cycle and free access to water and food. All procedures were carried out following the Care and Use of Laboratory Animals (license number: 2022D019).

### 2.3. Cell culture and the *in situ* malignant CRC mouse model

CT26 cells (CVCL_7254) were cultured in RPMI 1640 medium supplemented with 10% FBS and 1% penicillin and streptomycin (PS) and were incubated at 37°C in 5% CO_2_. The cells were subcultured in trypsin-EDTA (0.25%), and a single-cell suspension was obtained at a concentration of 10^7^ cells/mL. The suspension was injected into the left axilla of four mice in a volume of 100 μL per mouse. Subcutaneous tumors of about 1 cm in diameter grew after 10 days. The subcutaneous tumor-bearing mice were euthanized, their skin was disinfected, and the subcutaneous tumor was peeled off, and immediately immersed in saline containing 100 U/mL PS. The flesh-like tissues of the actively growing tumors were cut into 1 mm^3^ pieces.

The mice were randomly divided into the control group (CTL group, *n* = 5), the colonic tumor model group (CRC group, *n* = 7), and the sham surgery group (SHAM group, *n* = 5). Mice in the CRC group were anesthetized with inhaled isoflurane and placed in the supine position. An incision of about 1 cm was made in the center of the abdomen, and the cecum was carefully removed. The local plasma membrane at the junction between the cecum and colon was scraped off with a needle, a prepared tumor block was glued into the scratch with 3 M medical tissue glue, the external intestinal segment was backfilled into the abdominal cavity, and the wound was sutured. The entire process was performed aseptically. The mice were routinely housed in an SPF-grade animal laboratory for 15 days after awakening.

### 2.4. *Akkermansia muciniphila* culture and the over-colonized *Akkermansia muciniphila* mouse model

Liquid cultures of *A. muciniphila* for oral gavage were grown in fluid thioglycollate medium in an anaerobic chamber. The 5–7 days cultures were harvested, centrifuged at 4,000 × *g* for 10 min, and resuspended in sterile saline.

Fifteen mice were randomly divided into three groups (*n* = 5) of the control group (CTL group), the antibiotic cocktail group (1 g/L ampicillin, 1 g/L metronidazole, 0.5 g/L vancomycin, and 0.5 g/L neomycin, AMVN group), and the *A. muciniphila* + antibiotic cocktail group (AKK group). Ten mice were orally treated once daily for 2 days with 200 μL of an antibiotic cocktail before transplant. They were randomly assigned to the AMVN group or the AKK group. Mice in the AKK group were administered 400 μL of *A. muciniphila* (1 × 10^9^ CFU in sterile saline) once daily for 7 days, while mice in the CTL and AMVN groups were given equal doses of sterile saline. The weights of the mice were recorded daily. Fecal samples, blood, and serum were collected on the day after the last gavage. Then, all mice were humanly euthanized, and the colonic and fecal samples were collected. The stool, partial colonic tissue, and serum were stored at −80°C and the blood was stored at 4°C until analysis. Partial colonic segments were stored in tissue fixative (Wuhan Servicebio Technology Co., Ltd.) at 4°C for further procedures.

### 2.5. Sectioning and staining of the colonic tissue

The intact colon was removed from the euthanized mice, and a 1 cm section of colonic tissue was excised about 2 cm from the anus and placed in 4% paraformaldehyde for fixation. After the tissue was dehydrated in alcohol, it was hyalinized with xylene and embedded in paraffin. The embedded paraffin block was fixed and cut into 5–8 μm thick slices on a microtome and placed in a 45°C thermostat for drying.

The paraffin was removed from the sections with xylene, the hematoxylin and eosin (H&E) sections were washed with high to low concentrations of alcohol, and finally with distilled water before staining. The sections were placed in an aqueous hematoxylin solution for 3 min to stain the nuclei, and then in an eosin solution for 3 min to stain the cytoplasm. The sections were dehydrated with anhydrous ethanol and xylene, dried, and sealed with gum.

The colonic sections were stained with Alcian blue to detect the mucins. The colonic sections were dewaxed, dehydrated in gradient alcohol, and rehydrated in distilled water. The sections were soaked in an Alcian blue acidified solution for 3 min, stained with an Alcian blue staining solution for 30 min, and rinsed in running water for 5 min. The samples were re-stained in nuclear solid staining solution for 5 min and rinsed in running water for 1 min. The stained slides were scanned using Pannoramic SCAN (3DHISTECH Kft). Image analysis was performed with ImageJ.

### 2.6. Immunofluorescence analysis

The colonic sections were dewaxed and dehydrated in gradient alcohol. The sections were blocked with 2% BSA at 37°C for 30 min. Fluorescently labeled anti-ZO1 tight junction protein rabbit pAb (GB111402, Servicebio, Beijing, China) and anti-MUC2 rabbit pAb (GB11344, Servicebio) was added dropwise to the sections and incubated at 37°C for 30 min. The sections were rinsed three times with 0.01 mol/L PBS (pH = 7.4) for 5 min each time. Cell contours were formed using DAPI negative staining and blocked in buffered glycerol (analytically pure non-fluorescent glycerol mixed with pH = 9.2, 0.2 M carbonate buffer at 9:1). The stained slides were scanned as indicated above. Image analysis was performed with ImageJ.

### 2.7. Fecal DNA extraction and 16S ribosomal DNA gene sequencing

Feces were collected under sterile conditions before the mice were euthanized. The DNA was extracted from frozen fresh feces using the Stool Genomic DNA Extraction Kit (D2700, Solarbio® Life Science) according to the manufacturer’s instructions. The 16S ribosomal DNA (16S rDNA) gene sequencing method was described in our previous study (Huang et al., 2022). The V3–V4 16S rDNA target region of the ribosomal RNA gene was amplified by PCR (95°C for 5 min, followed by 30 cycles at 95°C for 1 min, 60°C for 1 min, and 72°C for 1 min and a final extension at 72°C for 7 min) using the forward primer 341F 5′-CCTACGGGNGGCWGCAG-3′ and the reverse primer 806R 5′-GGACTACHVGGGTATCTAAT-3′ (amplicon size was 466). PCR reagents were from New England Biolabs, United States.

The amplicons were extracted and purified from 2% agarose gels using the AxyPrep DNA Gel Extraction Kit (Axygen Biosciences, Union City, CA, United States) according to the manufacturer’s instructions. Then, those amplicons were quantified using ABI StepOnePlus Real-Time PCR System (Life Technologies, Foster City, United States). The purified amplicons were equimolar pooled and paired-end sequenced (PE250) on the Illumina NovaSeq 6000 according to the standard protocol (*n* = 5/group).

### 2.8. Quantitative real-time PCR analysis

The steps for fecal DNA extraction are described in Section 2.7. The DNA concentrations were determined by a spectrophotometer (NanoDrop, Thermo Fisher, Waltham, MA, United States). Quantitative real-time PCR was used to quantify the bacteria. Quantitative real-time PCR was performed with the 2X SG Fast qPCR Master Mix (High Rox; B639273, Beyotime, Beijing, China), and relative DNA expression was measured and analyzed with the ABI QuantStudio 6 Flex (Thermo Fisher). Each reaction was performed in triplicate. The 2^−ΔΔCt^ method was used to calculate the relative quantity of DNA compared to the internal control. Eubacteria was used as the internal reference gene. The final results of the AMVN and AKK groups were calculated relative to the CTL group. The relative primer sequences are available in Supplementary Table 1 (*n* = 3/group).

### 2.9. RNA extraction and quantitative reverse transcription PCR analysis

RNA was extracted from frozen colonic tissue using the RNAeasy™ Animal RNA Isolation Kit and a Spin Column (R0027, Beyotime) according to the manufacturer’s instructions. The contents of occludin, claudin-4, and ZO-1 mRNA were measured by quantitative reverse transcription PCR. Quantitative reverse transcription PCR was performed using the BeyoFast™ SYBR Green One-Step qRT-PCR Kit (D7268S, Beyotime), and relative RNA expression was measured and analyzed using the ABI QuantStudio 6 Flex (Thermo Fisher). Each reaction was performed in triplicate. The relative amount of RNA compared to the internal control was calculated by the 2^−ΔΔCt^ method. GAPDH was used as the internal reference gene. The relative primer sequences are available in Supplementary Table 1 (*n* = 4 for the AMVN group and *n* = 5 for the AKK group).

### 2.10. Data preprocessing and bioinformatics analysis

The 16S rDNA gene sequencing data were processed and analyzed by referencing our previous study (Huang et al., 2022). First, we used FASTP for filtering to obtain high-quality reads from the raw sequencing data. Then, the paired-end clean reads were merged as raw tags using FLASH (version 1.2.11) with a minimum overlap of 10 bp and a mismatch error rate of 2%.

The clean reads retained after quality control were clustered into operational taxonomic units (OTUs) of ≥97% similarity using the UPARSE (version 9.2.64) pipeline. All chimeric tags were removed using the UCHIME algorithm, and effective tags were obtained for further analysis. The tag sequence with the highest abundance was selected as the representative sequence within each cluster. The representative OTU sequences were classified into organisms using a naive Bayesian model and RDP classifier (version 2.2) based on the SILVA database (version 132) with a confidence threshold value of 0.8.

Alpha diversity indices were calculated using Usearch (version 10.0.240) and visualized using GraphPad Prism 9 (version 0.1.1; GraphPad Software Inc., La Jolla, CA, United States). The Vegan package (version 2.5–7) in R (version 4.0.5; The R Foundation for Statistical Computing, Vienna, Austria) was used to depict the Bray-Curtis distances for non-metric multidimensional scaling (NMDS). The graph of the NMDS plot and the stacked bar plots of the microbiota composition were visualized in R using the ggplot2 package (version 3.5.5). The unweighted pair group method with the arithmetic means (UPGMA) function in the R Vegan package was used to obtain the hierarchical clusters among the samples. The biomarker features were screened in each group using linear discriminant analysis effect size (LEfSe) analysis and LEfSe software (version 1.0).

### 2.11. Statistical analysis

All data were analyzed and graphs were prepared using GraphPad Prism 8.0. Two groups of data were analyzed using Student’s *t*-test and Welch’s *t*-test. Two-way ANOVA followed by Tukey’s multiple comparison test was used to analyze the data from more than two groups. Finally, the data were presented as mean ± SEM, and values of *p* < 0.05 were considered significant.

## 3. Results

### 3.1. Malignant damage of the colonic tissue in the *in situ* malignant CRC model

**Figure 1:**
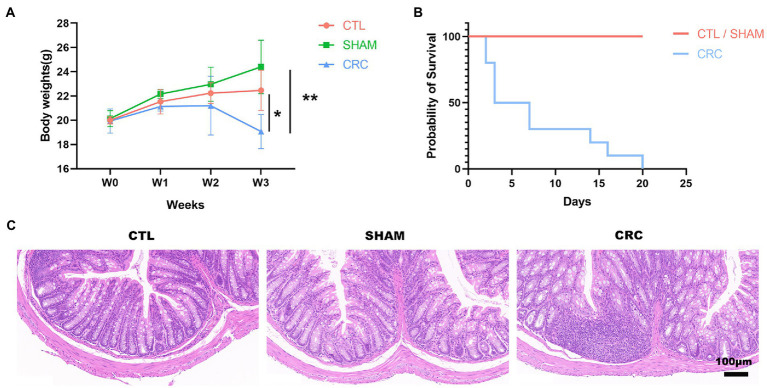
Malignant damage to the colonic tissue in the *in situ* malignant colorectal cancer (CRC) model. **(A)** Changes in the body weights of mice over 3 weeks. **(B)** Survival rate. **(C)** Hematoxylin and eosin (H&E) staining at W3. Scale bar, 100 μm. Data are mean ± SEM; *n* ≥ 3, ^*^p < 0.05, ^**^p < 0.005.

An *in situ* malignant CRC model was used to study the changes in the intestinal microenvironment in the cancer state. To assess the development of CRC, we examined the body weight and survival rate of the mice for 3 weeks. The mice were humanely euthanized in W3, and the colorectal tissue was collected for H&E staining to demonstrate intestinal damage. The body weight of the mice in the CRC group was significantly lower than that of the other groups (Figure 1A). Some mice in the CRC group died from tumors (Figure 1B). H&E staining displayed immune cell infiltration and colonic tissue damage. As shown in Figure 1C, the *in situ* tumors severely damaged the colonic tissue. A decrease in the number of intestinal glands in the intrinsic layer, a disorganized and distorted arrangement of the crypts, an increase in the number of basal plasma cells, and inflammatory infiltration were observed in the CRC group compared with the CTL and SHAM groups.

### 3.2. Decreased gut microenvironment abundance in the *in situ* malignant CRC model

**Figure 2:**
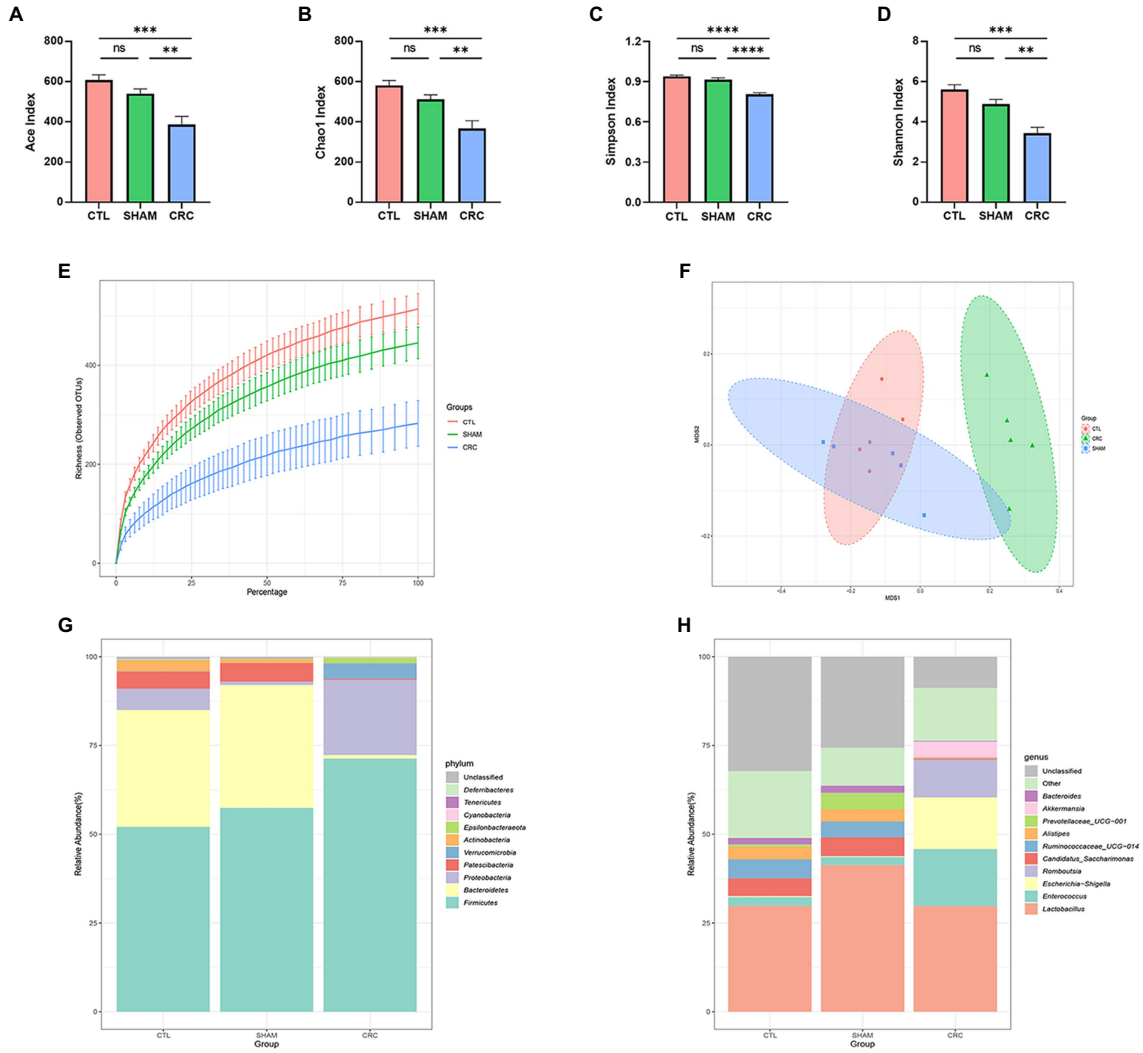
Decreased abundance of intestinal microbiota in CRC mice. Alpha diversity analysis of the three groups: Ace index **(A)**, Chao1 index **(B)**, Simpson index **(C)**, Shannon index **(D)**, and the richness dilution curve **(E)**. **(F)** Non-metric multidimensional scaling (NMDS) based on the operational taxonomic units (OTUs) in the three groups. Each point on the graph represents a sample. **(G,H)** Structural changes in the intestinal microbiota of the CRC model mice. The relative abundance of the top 10 microbial taxa was assessed at the **(G)** phylum and **(H)** generic levels. Data are mean ± SEM; *n* = 5, “ns,” not significant, ^**^p < 0.005, ^***^p < 0.0005, and ^****^p < 0.0001.

To investigate variations in the intestinal microenvironment of the mice, feces were collected during W3 to perform 16S rDNA sequencing. Intestinal microbiota diversity indicates the stability of the micro-ecology of the microbiota and its resistance to invasion by external pathogens (He et al., 2021). Alpha diversity was analyzed in multiple dimensions (dilution curves), perspectives (different indices), and forms, including the Ace index, the Chao1 index, the Simpson index, the Shannon index, and richness (Figures 2A–E). Higher alpha diversity indices indicate higher community diversity. The Ace index (Figure 2A), Chao1 index (Figure 2B), Simpson index (Figure 2C), Shannon index (Figure 2D), and richness (Figure 2E) were significantly lower in the CRC group than those in the CTL and SHAM groups. The gradual flattening of the dilution curves in all groups indicated that the number of species in the gut did not increase with the number of sequences. Therefore, the number of samples measured in this experiment was sufficient to characterize the microbiota. The results indicate that the abundance of the intestinal microbiota decreased significantly in mice with CRC and the stability of the intestinal microenvironment was disrupted.

We used NMDS and UPGMA clustering analysis to examine the structural differences among the three groups of samples. NMDS analysis reflects samples as points in a multidimensional space based on species information. The NMDS scatter plot (Figure 2F) reflected the discrepancies between the three groups of samples based on the distance between points. The 2D stress value was 0.06, which was well represented. NMDS analysis showed that the composition of the intestinal microenvironment in the CRC group was significantly distinct from that of the CTL and SHAM groups, suggesting that the gut microbial composition changed in mice due to CRC. The samples were classified in the UPGMA classification tree based on the beta diversity distance matrix information. Similar samples had fewer common branches. UPGMA clustering analysis (Supplementary Figure 1) showed similar findings as the NMDS analysis.

### 3.3. Structural changes in the intestinal microenvironment of the *in situ* malignant CRC model

The changes in the composition of the intestinal microbiota in CRC mice were further investigated. We analyzed the community structure of the entire sample at the phylum level. The relative abundances of the top 10 microbial taxa are reported as stacked histograms (Figure 2G). The relative abundances of *Bacteroidetes*, *Patescibacteria*, and *Actinobacteria* decreased in the CRC group, while the relative abundances of *Firmicutes*, *Proteobacteria*, *Verrucomicrobia*, and *Epsilonbacteraeota* increased compared with those in the CTL and SHAM groups. The relative abundance results at the phylum level demonstrated that the intestinal microbiota composition was altered in the CRC mice.

To further reveal the changes at the genus level, the relative abundance results of the top 10 genera are shown in Figure 2H. The structural variations in the CRC group were significant compared to those in the CTL and SHAM groups. CRC decreased the abundances of *Candidatus saccharimonas*, *Ruminococcaceae UCG-014*, *Alistipes,* and *Bacteroides*, while it significantly increased the abundances of *Enterococcus*, *Escherichia shigella*, *Romboutsia*, and *Akkermansia*. Similarly, the structure of the intestinal microbiota was significantly altered in the CRC group and homeostasis of the intestinal microenvironment was disrupted.

### 3.4. Identification of the signature bacteria in CRC and excess colonization of *Akkermansia muciniphila*

**Figure 3:**
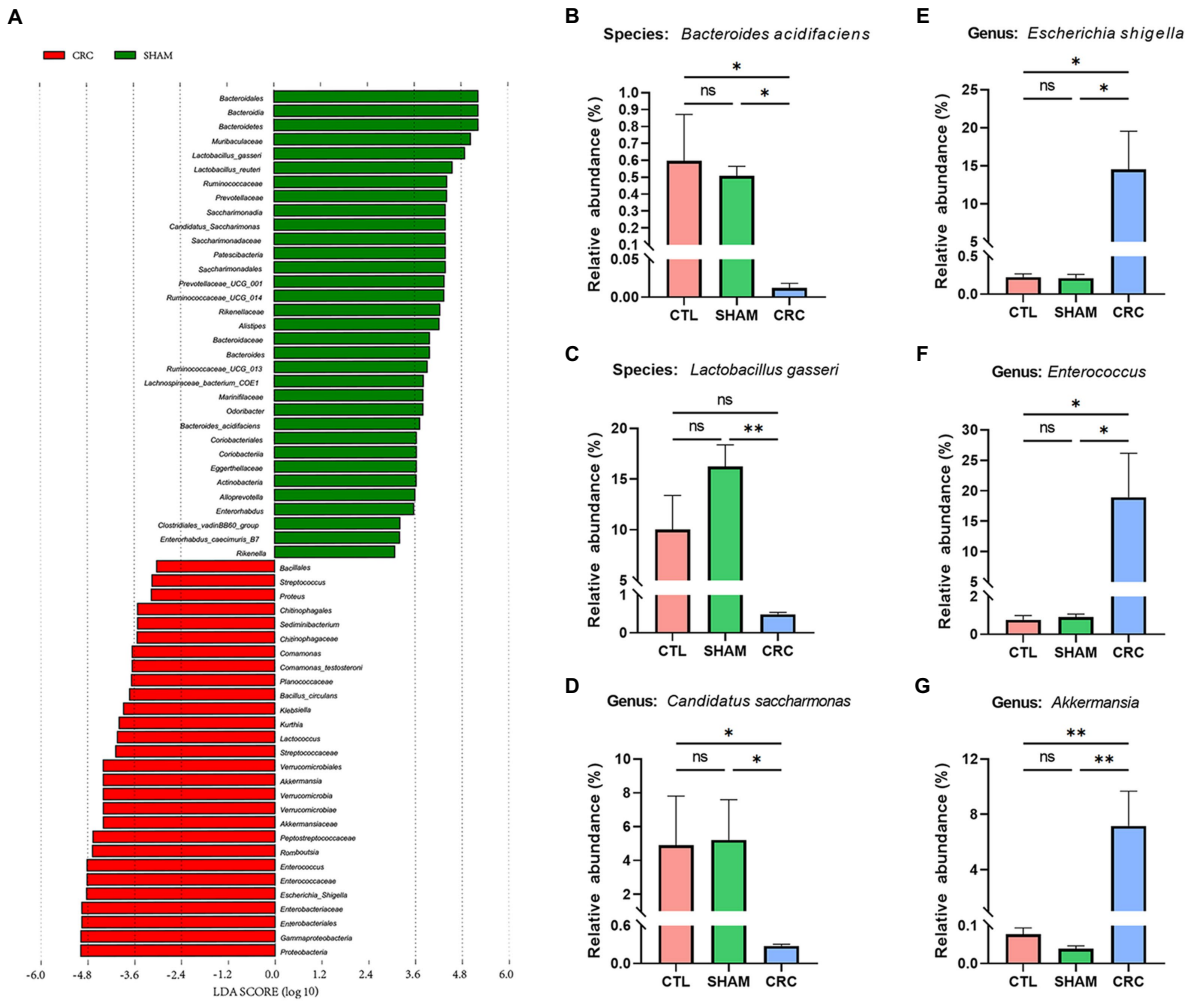
Identification of signature bacteria in CRC and excess colonization of *Akkermansia muciniphila*. **(A)** Linear discriminant analysis effect size (LEfSe) analysis between the CRC and SHAM groups with the highest linear discriminant analysis (LDA) score [log (LDA score) ≥ 3]. Relative abundances of *Bacteroides acidifaciens* **(B)**, *Lactobacillus gasseri* **(C)**, *Candidatus saccharimonas* **(D)**, *Escherichia Shigella* **(E)**, *Enterococcus* **(F)**, and *Akkermansia* **(G)**. Data are mean ± SEM; *n* ≥ 3, “ns,” not significant, ^*^p < 0.05, and ^**^p < 0.005.

We used LEfSe software to count significantly different biomarkers in the CRC and SHAM groups and further identify the key microbiota in the guts of the CRC mice. Linear discriminant analysis (LDA) effects were used to identify characteristic biomarkers (Figure 3A). The logarithmic LDA score for significant differences was set to 3, and the corresponding cladogram is provided in Supplementary Figure 2. *Bacteroides acidifaciens*, *Lactobacillus gasseri*, *Ruminococcaceae*, *Prevotellaceae*, and *C. saccharimonas* were enriched in the SHAM group. However, *E. Shigella* from *Enterobacteriaceae*, *Enterococcus*, *Romboutsia*, *Lactococcus*, and *Akkermansia* were enriched in the CRC group.

The relative abundance statistics of the characteristic microbiota were further analyzed to determine the environmental differences among the three groups. The relative abundances of *B. acidifaciens* (Figure 3B), *L. gasseri* (Figure 3C), and *C. saccharimonas* (Figure 3D) decreased significantly in the CRC group. In contrast, the relative abundances of pathogenic bacteria, such as *E. Shigella* (Figure 3E) and *Enterococcus* (Figure 3F), increased. Notably, the relative abundance of *Akkermansia* increased compared to that in the CTL and SHAM groups (Figure 3G).

We repeated the same experiments to examine the changes in the gut microbiota in the CRC model. The gut microbiota of the CRC group was significantly different from that of the control group, as shown in Supplementary Figures 3A–E. Significant differences were observed at the phylum and genus levels, and the relative abundances of *Verrucomicrobia* and *Akkermansia* increased significantly in the CRC group (Supplementary Figures 3F,G). The LEfSe analysis also showed that *Akkermansia* was an extremely significantly enriched bacterial species in the model group (Supplementary Figure 3H). These results suggest that the relative abundance of *Akkermansia* in the intestines of CRC mice increased significantly. These results reveal the reproducibility of the aforementioned experiments.

### 3.5. Excess colonization of *Akkermansia muciniphila* leads to minor inflammation

**Figure 4:**
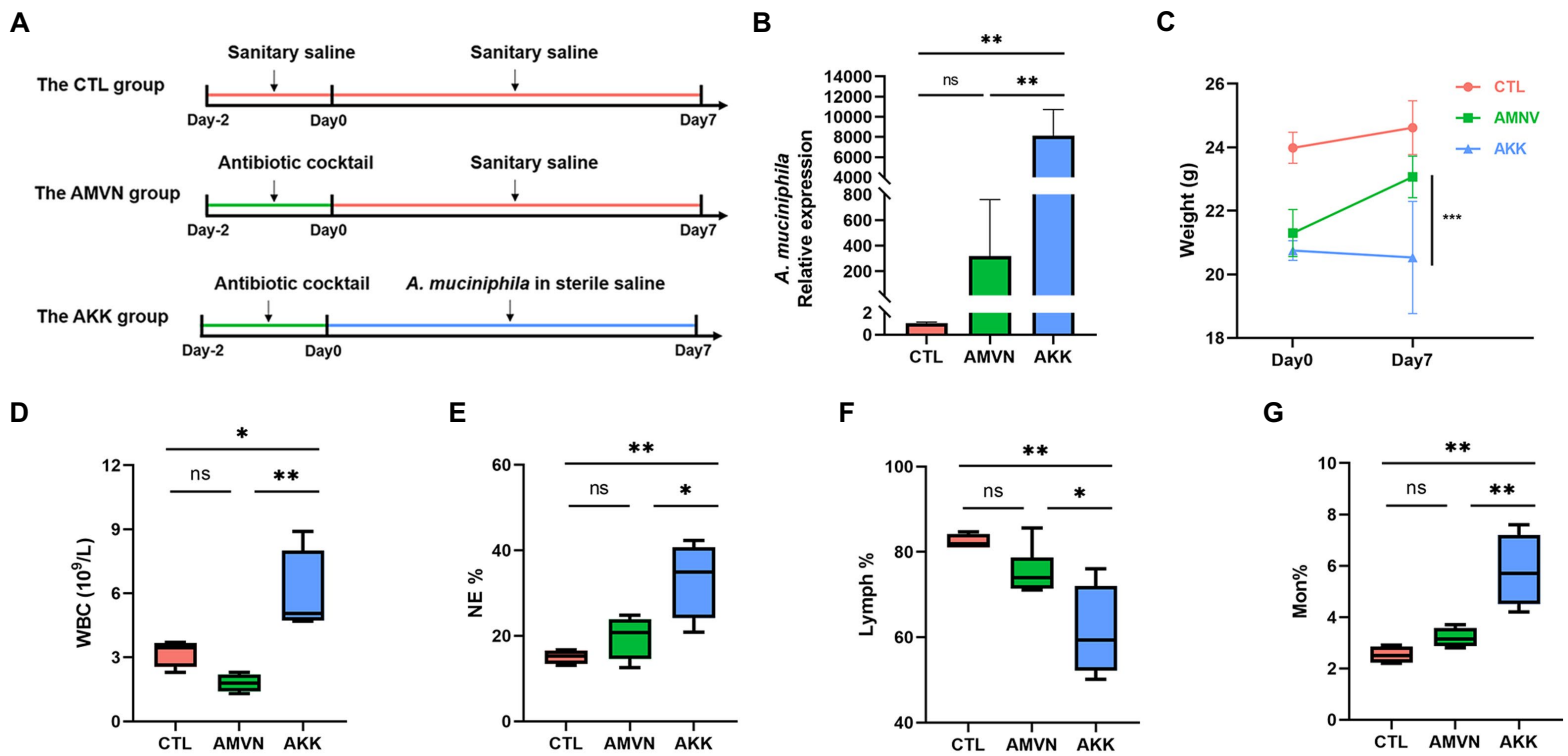
Excess colonization of the *Akkermansia muciniphila* mouse model. **(A)** Schematic diagram of the experiment. **(B)** Relative expression of *A. muciniphila* in mice feces tested by qRT-PCR. **(C)** Body weights before and after administering *A. muciniphila*. **(D)** The quantity of white blood cells (WBC). The percentages of neutrophils (NE) **(E)**, lymphocytes (Lymph) **(F)**, and monocytes (Mon) **(G)**. Data are mean ± SEM; *n* ≥ 3, “ns,” not significant, ^*^p < 0.05, ^**^p < 0.005, and ^***^p < 0.0005.

The over-colonized *A. muciniphila* mouse model was established by gastric gavage with live *A. muciniphila* after the antibiotic treatment to assess the condition of the intestinal barrier during *A. muciniphila* over-colonization (Figure 4A). The feces of the mice were collected on the final day, and colonization by *A. muciniphila* was detected by qRT-PCR. As shown in Figure 4B, the *A. muciniphila* mRNA relative expression level in the AKK group was significantly higher than that in the CTL and AMVN groups. To show the role of antibiotic in *A. muciniphila* over-colonization, we added a group (AKK^−^) with the same concentration of *A. muciniphila* gavage but no antibiotic pretreatment. The results indicated no significant difference in the relative expression of *A. muciniphila* between the AKK^−^ and CTL groups (Supplementary Figure 4). However, the relative expression of *A. muciniphila* was significantly higher in the AKK group than in the AKK-group. These results show that the antibiotic cocktail treatment caused dysregulation of the intestinal microbiota, which aided *A. muciniphila* colonization and the over-colonized *A. muciniphila* mouse model was successfully established. Figure 4C shows the body weights of the mice before and after the *A. muciniphila* gavage. The body weights of the AKK and AMVN groups were lower on day 0 due to the antibiotic treatment compared to the CTL group. Mice in the AKK group had significantly lower body weights.

**Figure 5:**
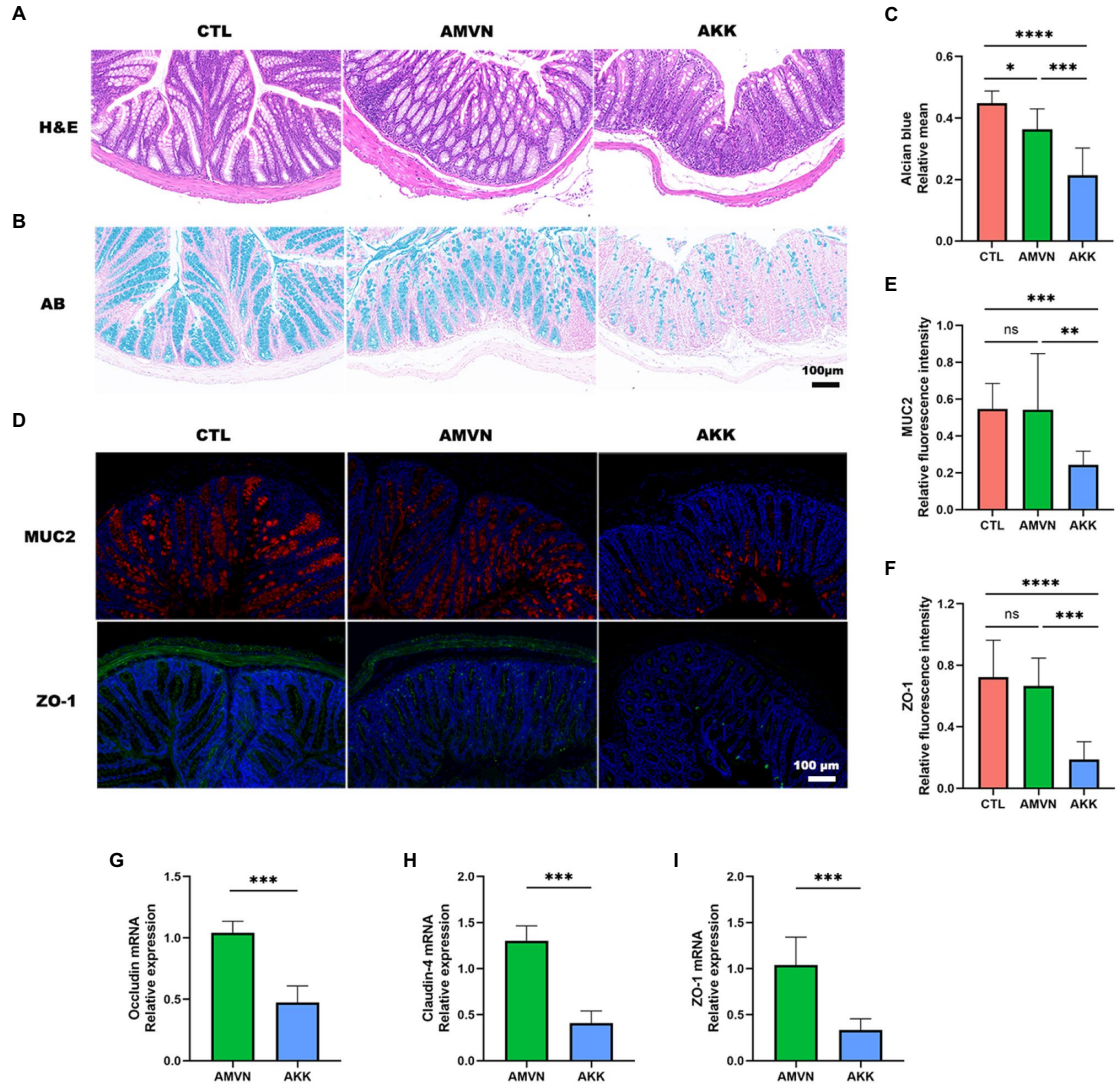
Disruption of the intestinal barrier by excess colonization of *Akkermansia muciniphila.* **(A)** H&E staining of colonic tissues. Scale bar, 100 μm. **(B)** Alcian blue staining of colonic tissues. Scale bar, 100 μm. **(C)** Quantitative analysis of Alcian blue staining; relative mean. **(D)** Immunofluorescence staining of colon tissues for MUC2 (red) and ZO-1 (green). DAPI (blue) was used to stain nuclei. Scale bar, 100 μm. Quantitative analysis of MUC2 **(E)** and ZO-1 **(F)** relative fluorescence intensity. The relative mRNA expression of occludin **(G)**, claudin-4 **(H)**, and ZO-1 **(I)** in the intestinal tissue in the AKK and AMVN groups. Data are mean ± SEM; *n* ≥ 3, “ns,” not significant, ^*^p < 0.05, ^**^p < 0.005, ^***^p < 0.0005, and ^****^p < 0.0001.

We determined whether the over-colonization of *A. muciniphila* caused inflammation using hematological analysis. As shown in Figures 4D–G, the number of white blood cells (WBCs) in the AKK group increased significantly compared with that in the CTL and AMVN groups. After the mice were treated with *A. muciniphila*, the percentages of neutrophils (NE) and monocytes (Mon) increased, whereas the percentages of lymphocytes (Lymph) decreased. These results indicate that the excess colonization of *A. muciniphila* caused mild inflammation. H&E staining of the colonic tissues (Figure 5A) revealed that excess colonization of *A. muciniphila* destroyed crypts, caused edema, and resulted in massive inflammatory cell infiltration. This result was consistent with the hematological analysis. The excess colonization of *A. muciniphila* inflamed and destroyed the colonic tissue.

### 3.6. Disruption of the intestinal barrier by excess *Akkermansia muciniphila* colonization

The mucus layer is the first defense barrier against pathogens, and goblet cells secrete mucins to maintain the thickness of the mucus layer (Gustafsson and Johansson, 2022). To determine the effect of *A. muciniphila* over-colonization on the mucus layer, colonic tissues were stained with Alcian blue (goblet cells stain blue). As shown in Figure 5B, the CTL group exhibited regular goblet cell morphology and a normal mucus layer, while the AMVN group developed slight thinning of the mucus layer. Notably, the mucus layer in the colonic tissue of the AKK group was severely damaged and the number of goblet cells decreased significantly. The area of blue in the images was determined quantitatively to assess the degree of disruption of the mucus layer (Figure 5C). The relative mean of the Alcian blue-stained area was significantly lower in the AKK group than that in the CTL and AMVN groups.

Mucin 2 (MUC2) is a secreted mucin, and the main mucin in the colonic barrier, which plays a role in lubrication and protects the colonic mucosa against bacteria and toxins. The MUC2 level has been used as an important indicator of colonic mucosal integrity. ZO-1 is an indicator of the function of the tight junction barrier between intestinal epithelial cells. Thus, the expression levels of MUC2 and ZO-1 were detected in colonic tissues by immunofluorescence (Figure 5D). The MUC2 protein was red, ZO-1 was green, and DAPI was used to stain the cell nuclei. The relative fluorescence intensities of MUC2 and ZO-1 were statistically quantified among the three groups. As shown in Figures 5E,F, relative fluorescence intensity was significantly lower in the AKK group than that in the CTL and AMVN groups, indicating that the MUC2 and ZO-1 levels were low in the colonic tissue of the AKK group. In addition, the relative mRNA expression levels of occludin, claudin-4, and ZO-1 also decreased in the intestinal tissue (Figures 5G–I). These results suggest that excess colonization of *A. muciniphila* led to substantial catabolism of mucin and tight junction proteins in colonic tissues and an impaired intestinal barrier.

## 4. Discussion

In this study, we prepared an *in situ* malignant CRC model by transplanting tumor tissues from subcutaneous tumors created with CT26 cells. Then, we examined the changes in intestinal microbial ecology and composition in CRC mice using 16S rDNA sequencing. Remarkably, the relative abundance of *A. muciniphila* increased at the genus level in the LDA analysis. In our recent study, we noticed a similar over-colonization in mice with DSS-induced ulcerative colitis (Huang et al., 2022). As mentioned before, the characteristics of *A. muciniphila* are related to the mucus layer of the intestinal barrier. Therefore, we established an over-colonized *A. muciniphila* mouse model to confirm whether the role of *A. muciniphila* in intestinal barrier damage is compensatory regulation or aggravation. The level of intestinal barrier-related mucins and tight junction proteins decreased in intestinal tissues, confirming that the over-colonized *A. muciniphila* caused intestinal barrier damage.

In our study, we established the over-colonized *A. muciniphila* mouse model by gastric gavage with live *A. muciniphila* (1 × 10^9^ CFU) after the antibiotic treatment. Some studies (Everard et al., 2013; Plovier et al., 2017) have indicated that *A. muciniphila* (2 × 10^8^ CFU) gavage alone has no detrimental effect. Notably, the antibiotic cocktail was an important factor contributing to colonization by *A. muciniphila*. As the same time, we used the *Eubacteria* primers as an internal reference for total bacteria (or as the housekeeping gene). These primers are universal eubacteria community primers (UniF340 and UniR514), which represent all bacteria and are widely used in qRT-PCR and 16S rDNA studies to determine bacterial abundance or capture the 16S rDNA of all bacteria (Wang et al., 1996; Lawley et al., 2017; Kuhbandner et al., 2019; Chen et al., 2022). The target threshold cycle (Ct) for the relative qRT-PCR expression results was divided by the internal reference Ct using the delta–delta Ct method. Thus, our qRT-PCR results reflect the relative amount of *A. muciniphila* to the overall microbiota. The abundance of *A. muciniphila* in the AKK group was significantly higher than that in the CTL and AMVN groups, so *A. muciniphila* was over-colonized.

The intestinal mucus barrier is the first natural barrier against the invasion of harmful substances. It plays an active role defending against the invasion of disease-causing microorganisms and assisting in the colonization of probiotics (Johansson et al., 2008). MUC2 is a heavily glycosylated secretory mucin secreted by intestinal goblet cells and is the main component of the intestinal mucus barrier (Yao et al., 2021). *Akkermansia muciniphila* normally colonizes the outer layer of loose mucus and upregulates the synthesis of MUC2 by goblet cells through metabolites (Meng et al., 2020). Although *A. muciniphila* degrades the mucus layer, it does not change its thickness, which reaches a dynamic equilibrium (Derrien et al., 2017). In our study, over-colonization of *A. muciniphila* resulted in a significantly lower MUC2 level than normal, suggesting that over-colonized *A. muciniphila* consumes more mucin than goblet cells secrete. As a result, both the intestinal mucus barrier and the balance of the intestinal barrier were compromised.

The primary component of the intestinal barrier is intestinal mucosal epithelial cells, which are held together by tight junction proteins. The mechanical integrity and proper operation of the intestinal mucosal barrier are maintained by these tight junctions (Suzuki, 2020). Tight junctions consist of occludin, claudins, junction adhesion molecules, and peripheral cytoplasmic proteins, such as ZO-1, ZO-2, and ZO-3. Our study revealed that ZO-1 content decreased significantly in the colonic tissue of the over-colonized *A. muciniphila* mouse model, and the relative mRNA expression levels of occludin, claudin-4, and ZO-1 also decreased in the intestinal tissue. As shown in our results, an overabundance of *A. muciniphila* led to a thinning of the intestinal mucus layer, which allowed harmful bacteria to directly contact the intestinal epithelial cells, further causing epithelial cell damage and disrupting the tight junctions between the cells. At the same time, the persistent deficiency of mucins and the difficulties in repairing the intestinal mucus layer were accompanied by further weakening of the repair capacity of the intestinal epithelial cells. These two factors contributed to disrupt the intestinal barrier and aggravate the development of colitis and CRC. These factors also contributed to bacterial translocation, providing opportunities for pathogenic bacteria and potentially carcinogenic metabolites. Harmful substances can access the peripheral circulation, causing systemic tissue damage and possibly leading to serious autoimmune diseases, such as type 1 diabetes (Costa et al., 2016) or systemic lupus erythematosus (Ogunrinde et al., 2019).

*Akkermansia muciniphila* is widely considered to be a next-generation probiotic. Clinical trial results have shown that pasteurized *A. muciniphila* has a positive effect on overweight and obese human volunteers rather than live *A. muciniphila* (Depommier et al., 2019). Many conflicting results about the effects of *A. muciniphila* have been reported by IBD-related studies (Zhang et al., 2021). It has also been shown that *A. muciniphila* exerts anti-inflammatory effects through extracellular vesicles (Kang et al., 2013; Chelakkot et al., 2018). However, one study revealed that *A. muciniphila* interferes with mucosal reconstitution and exacerbates inflammation in *Salmonella enterica typhimurium*-induced gut inflammation (Ganesh et al., 2013). This may be due to *A. muciniphila-*enhanced catabolism of mucin products, which provides other gut pathogenic bacteria the substances they needed to grow. Another study found that the abundance of *A. muciniphila* increased significantly in the mouse intestines after worsening of spontaneous colitis caused by NLRP6 gene knockout in IL-10^−/−^ mice (Seregin et al., 2017), and that *A. muciniphila* administration orally worsened the colitis. It has been demonstrated that the abundance of *A. muciniphila* is strongly associated with Parkinson’s disease and is a disease signature microbiota (Hill-Burns et al., 2017; Heintz-Buschart et al., 2018). A higher abundance of *A. muciniphila* has also been observed in multiple sclerosis and Alzheimer’s disease (Berer et al., 2017; Vogt et al., 2017). Although the causality and mechanisms associated with these observations remain unclear, these findings suggest that *A. muciniphila* may be a double-edged sword.

Our findings support the idea that intestinal *A. muciniphila*, which also be considered as probiotics, may have negative effects in certain pathological situations and aggravate intestinal disease symptoms. *Akkermansia muciniphila* becomes a potential pathogenic agent under some conditions. Thus, it is crucial to analyze the function of *A. muciniphila* in many contexts before use in clinical treatment. Our findings raise awareness of the possible pathogenic role of live *A. muciniphila* as a probiotic. Live *A. muciniphila* aggravates damage to the intestinal barrier and promotes the development of inflammation and cancer. Thus, it is not possible to use *A. muciniphila* to treat patients with diseases, such as colitis and CRC.

However, *A. muciniphila* also affects the ability of intestinal goblet cells to secrete mucin; the mechanism underlying this effect remains unknown and requires further research. Furthermore, we did not test the effect of *A. muciniphila* on other gut microorganisms, and the interactions between *A. muciniphil*a and other bacteria were not determined. These limitations will be answered in our future studies. The findings of this study have important implications for understanding how *A. muciniphila* interferes with the reconstruction of a damaged intestinal mucus layer. They suggest that we must consider the patient’s condition when applying *A. muciniphila* to the clinic.

## 5. Conclusion

Our findings indicate that the abundance and structure of the gut microbiota are disturbed in CRC mice with substantial damage to the intestinal barrier. Notably, the number of *A. muciniphila* increased significantly in the context of intestinal disease. Excess *A. muciniphila* degraded mucins rather than increasing mucin synthesis in the intestinal tissues. Disruption of the tight junctions of intestinal epithelial cells and the ability to produce mucin to repair the mucosal barrier worsened the intestinal mucosal environment, which promotes the displacement of microbiota and exacerbates inflammation and cancer. Our study suggests that over-colonized *A. muciniphila* contributes to the genesis and progression of intestinal diseases by disrupting the intestinal barrier through excessive consumption of mucin.

## Data availability statement

The data presented in the study are deposited in the Genome Sequence Archive (Genomics, Proteomics and Bioinformatics 2021) in National Genomics Data Center (Nucleic Acids Res 2022), China National Center for Bioinformation/Beijing Institute of Genomics, Chinese Academy of Sciences (GSA: CRA009251) that are publicly accessible at https://ngdc.cncb.ac.cn/gsa/browse/CRA009251.

## Ethics statement

The animal study was reviewed and approved by Animal Ethics Committee of the Institute of Chinese Materia Medica.

## Author contributions

SQ, YZ, and YH contributed to conception and design of the study under the guidance of MQ, and KN. SQ, and YZ carried out most experiments. YH was responsible for the bioinformatic analysis. SQ and YZ wrote the first draft of the manuscript. YF, KX, WZ, and YW carried out article correction and provided guidance on the overall study. All authors contributed to the article and approved the submitted version.

## Funding

This work was partially or fully sponsored by the National Key Research and Development Program of China with grant no. ZK20200085.

## Conflict of interest

The authors declare that the research was conducted in the absence of any commercial or financial relationships that could be construed as a potential conflict of interest.

## Publisher’s note

All claims expressed in this article are solely those of the authors and do not necessarily represent those of their affiliated organizations, or those of the publisher, the editors and the reviewers. Any product that may be evaluated in this article, or claim that may be made by its manufacturer, is not guaranteed or endorsed by the publisher.

## Supplementary material

The Supplementary material for this article can be found online at: https://www.frontiersin.org/articles/10.3389/fmicb.2023.1111911/full#supplementary-material
