# Supplementary material for: Excessive consumption of mucin by over-colonized Akkermansia muciniphila promotes intestinal barrier damage during malignant intestinal environment
Source: Front Microbiol. 2023 Mar 2;14:1111911. doi: 10.3389/fmicb.2023.1111911 (PMC10018180; doi:10.3389/fmicb.2023.1111911)
Supplement: Supplementary file 1 [file Data_Sheet_1.docx]

Supplementary Material

Excessive consumption of mucin by over-colonized *Akkermansia muciniphila* promotes intestinal barrier damage during malignant intestinal environment

Shuang Qu^1†^, Yinghui Zheng^1†^, Yichun Huang^1†^, Yicheng Feng^1^, Kunyao Xu^1^, Wei Zhang^1^, Yawen Wang^1^, Kaili Nie^1*^, Meng Qin^1*^

*** Correspondence:**Meng Qin
[qinmeng212@mail.buct.edu.cn](mailto:qinmeng212@mail.buct.edu.cn)

Kaili Nie

[2012500071@mail.buct.edu.cn](mailto:2012500071@mail.buct.edu.cn)

^†^These authors have contributed equally to this work

**Supplementary Table 1** Primer sequences

| Primer | Primer sequences |
| --- | --- |
| *A. muciniphila* | F: 5’- CAGCACGTGAAGGTGGGGAC -3’ |
|  | R: 5’- CCTTGCGGTTGGCTTCAGAT -3’ |
| *Eubacteria* | F: 5’- ACTCCTACGGGAGGCAGCAGT -3’ |
|  | R: 5’- ATTACCGCGGCTGCTGGC -3’ |
| GAPDH | F: 5’- TGGCCTTCCGTGTTCCTAC -3’ |
|  | R: 5’- GAGTTGCTGTTGAAGTCGCA -3’ |
| occludin | F: 5’- TTCCTCTGACCTTGAGTGTGG -3’ |
|  | R: 5’- CTCTTGCCCTTTCCTGCTTT -3’ |
| claudin-4 | F: 5’- GTCCTGGGAATCTCCTTGGC -3’ |
|  | R: 5’- TCTGTGCCGTGACGATGTTG -3’ |
| ZO-1 | F: 5’- GCCGCTAAGAGCACAGCAA -3’ |
|  | R: 5’- GCCCTCCTTTTAACACATCAGA -3’ |

Abbreviation: *A. muciniphila*, *Akkermansia muciniphila*; GADPH, glyceraldehyde-3-phosphate dehydrogenase; ZO-1, zonula occludens-1


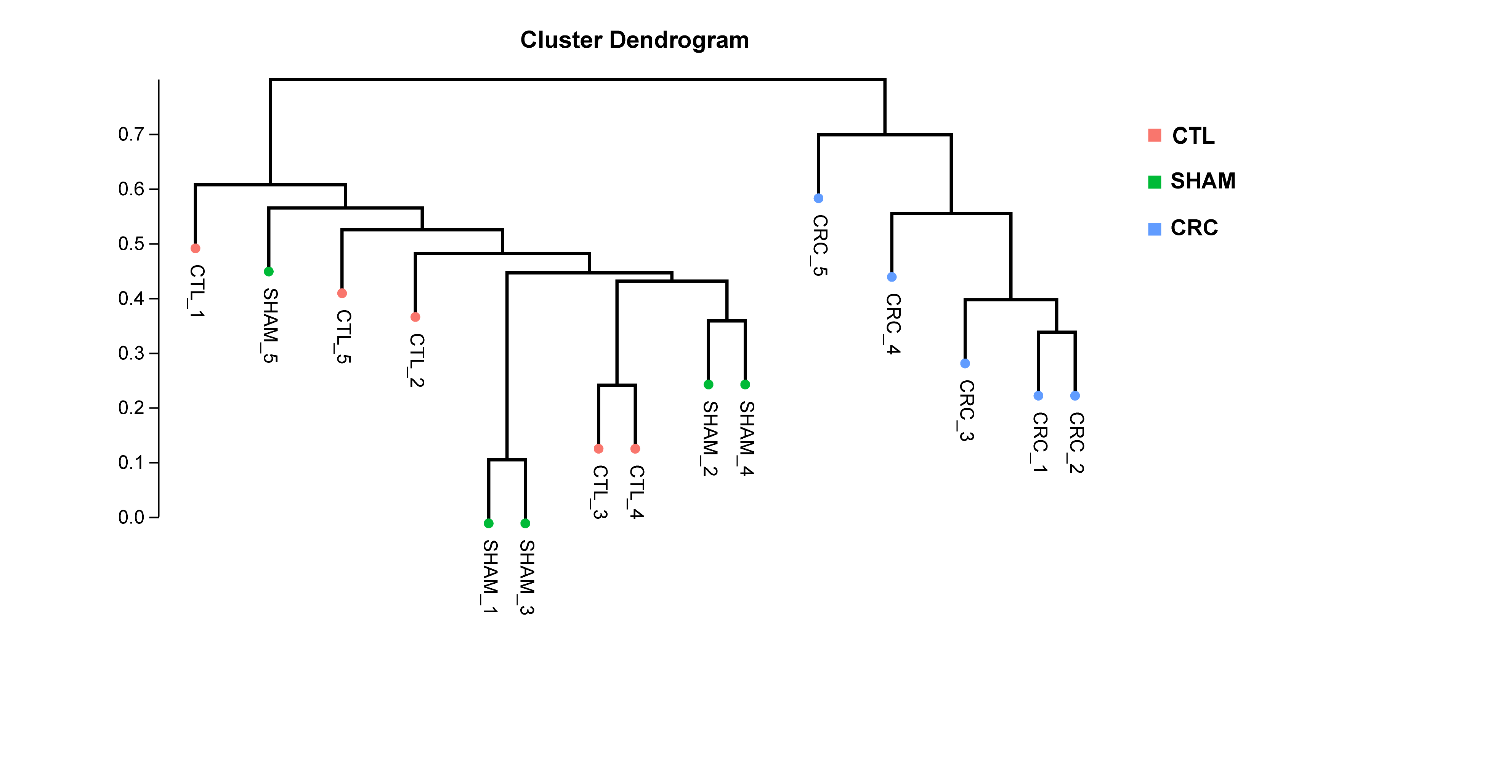


**Supplementary Figure 1.** The cluster dendrogram of UPGMA clustering analysis among the CTL group, the CRC group, and the SHAM group at W3.


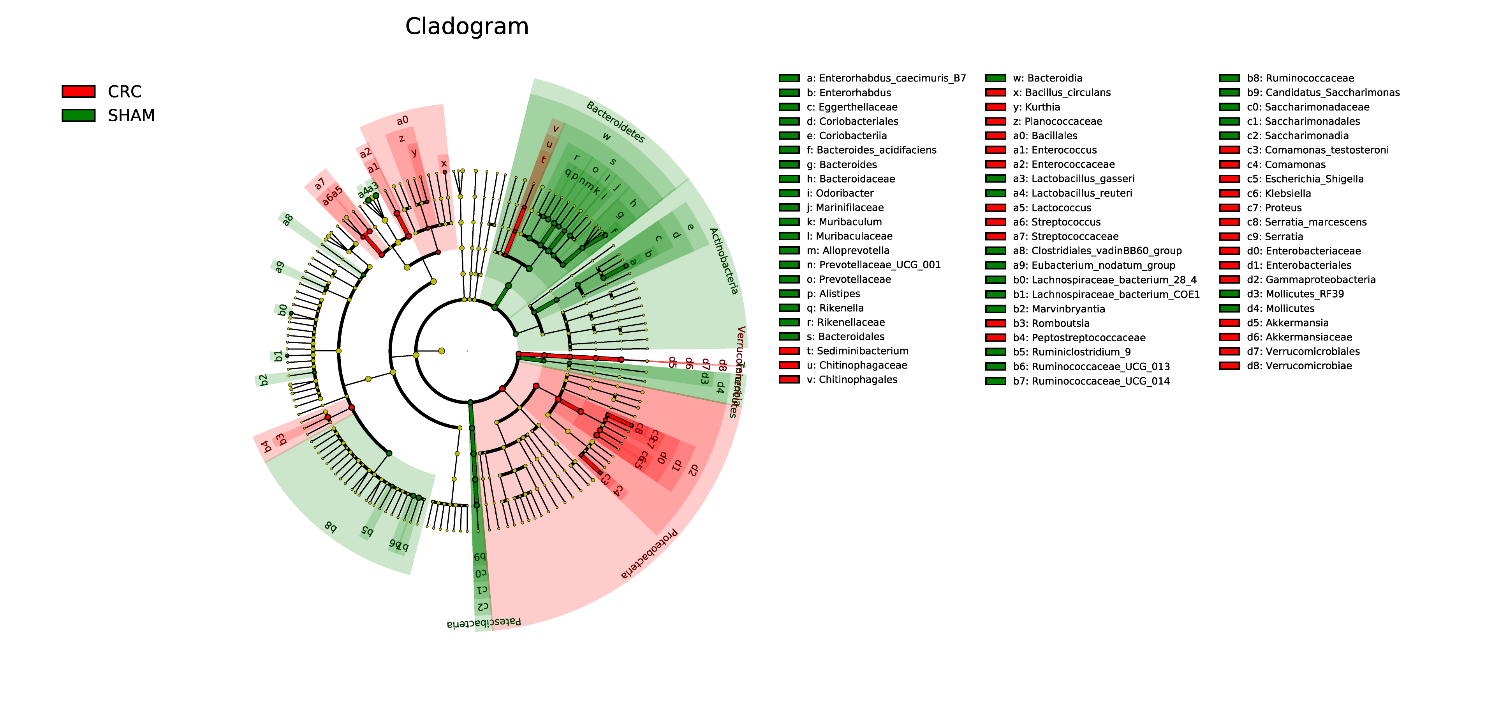


**Supplementary Figure 2.** The cladogram of LEfSe analysis among the CRC group and the SHAM group at W3.


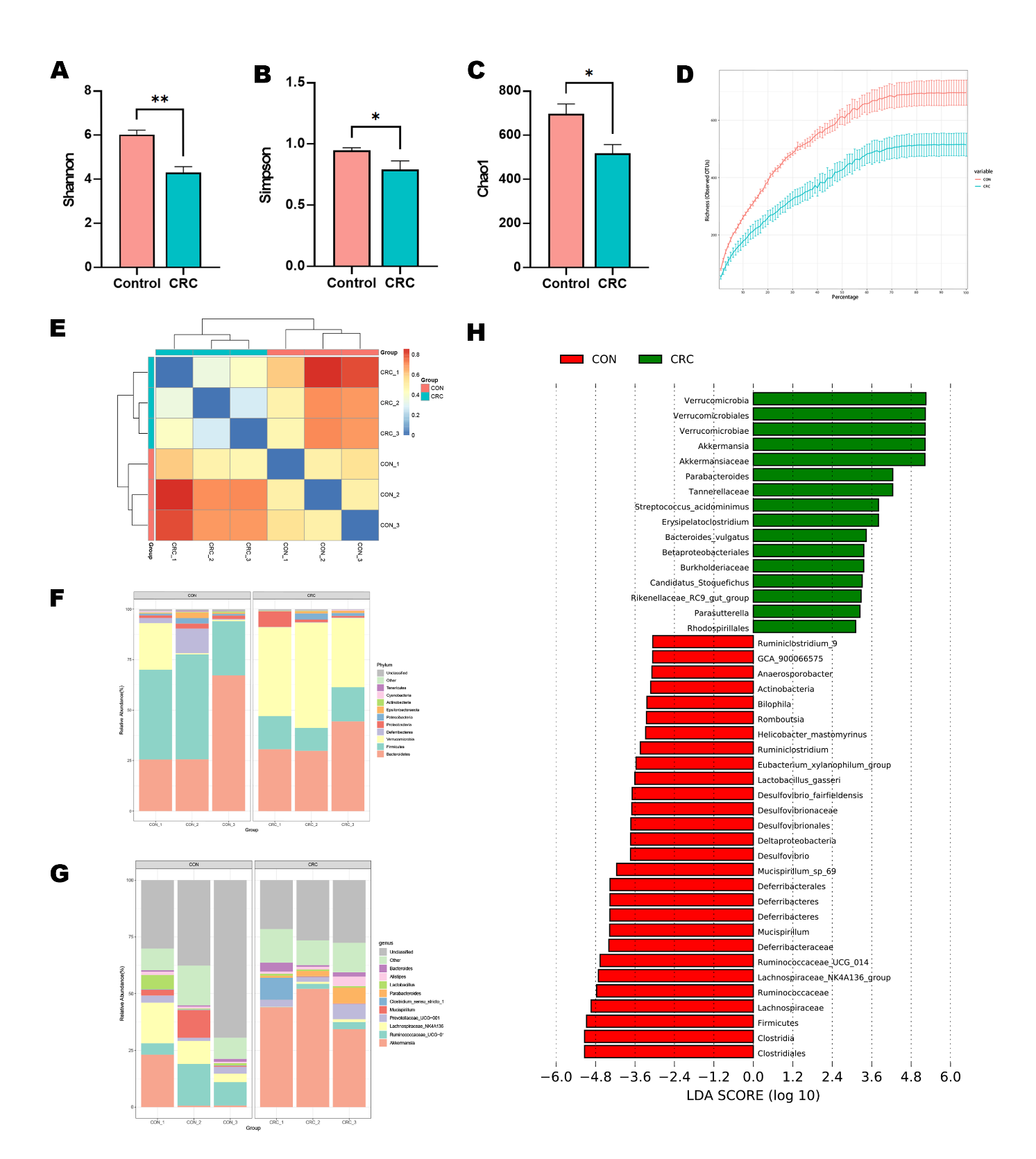


**Supplementary Figure 3.** Significant changes of gut microbiota in the CRC mice model. Alpha diversity analysis of two groups: Shannon index (A), Simpson index(B), Chao1 index(C), and the dilution curve of richness(D). (E) Bray-Curtis’s distance heatmap between CRC and control group samples. The relative abundance of the top 10 microbial taxa was assessed at the (F) phylum and (G) genus levels. (H)LEfSe analysis between the CRC group and the control group with the highest linear discriminant analysis (LDA) score (log (LDA score) ≥ 3). Data are mean ± SEM. n = 3, *p<0.05 and **p<0.005.


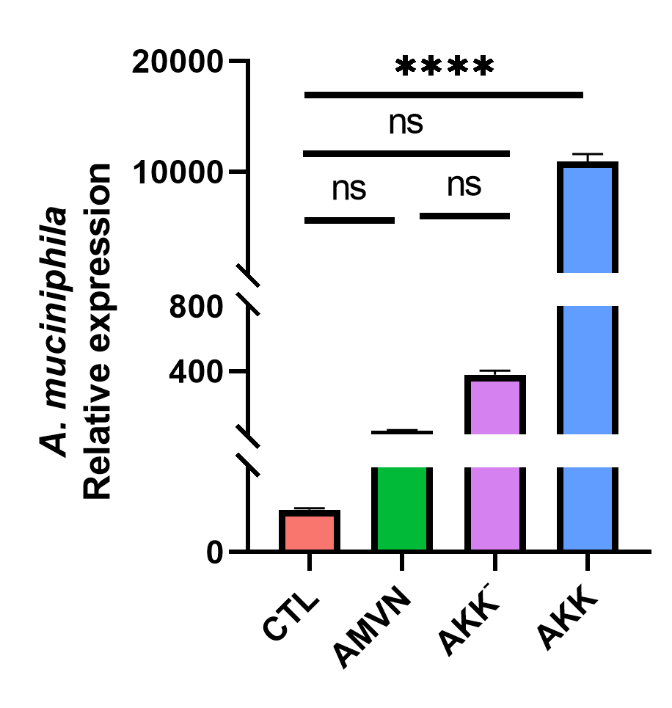


**Supplementary Figure 4.** *A. muciniphila* relative expression in mice faces tested by qRT-PCR. The control group (CTL), the antibiotic cocktail group (AMVN), the only *A. muciniphila* group (AKK^-^), and the antibiotic cocktail + *A. muciniphila* group (AKK). Data are mean ± SEM. “ns”, not significant, n = 4, ****p<0.0001.
